# 24-Epibrassinolide promotes NO_3_^−^ and NH_4_^+^ ion flux rate and NRT1 gene expression in cucumber under suboptimal root zone temperature

**DOI:** 10.1186/s12870-019-1838-3

**Published:** 2019-05-30

**Authors:** Ali Anwar, Yansu Li, Chaoxing He, Xianchang Yu

**Affiliations:** The Institute of Vegetables and Flowers, Chinese Academy of Agricultural Scieces, Beijing, China

**Keywords:** 24-Epibrassinolide, Root zone temperature, NO_3_^−^ and NH_4_^+^ flux rates, NRT1 genes

## Abstract

**Background:**

Suboptimal root zone temperature (RZT) causes a remarkable reduction in growth of horticultural crops during winter cultivation under greenhouse production. However, limited information is available on the effects of suboptimal RZT on nitrogen (N) metabolism in cucumber seedlings. The aim of this study is to investigate the effects of 24-Epibrassinolide (EBR) on nitrate and ammonium flux rate, N metabolism, and transcript levels of *NRT1* family genes under suboptimal RZT in cucumber seedlings.

**Results:**

Suboptimal RZT (LT) negatively affected on cucumber growth and proportionately decreased EBR contents, bleeding rate, root activity, enzyme activities of nitrate reductase (NR), nitrite reductase (NiR), glutamine synthetase (GS), and glutamate synthase (GOGAT), nitrate (NO_3_^−^) influx rate, ammonium (NH_4_^+^) efflux rate, and transcript levels of nitrate transporter (*NRT1*) encoding genes. However, exogenous EBR reduced the harmful effects of suboptimal RZT and increased endogenous EBR contents, bleeding rate, root activity, enzyme activities of NR, NiR, GS, and GOGAT, NH_4_^+^ and NO_3_^−^ flux rates and contents, and N accumulation. EBR-treated seedlings also upregulated the transcript levels of nitrate transporters *CsNRT1.1*, *CsNRT1.2A*, *CsNRT1.2B*, *CsNRT1.2C*, *CsNRT1.3*, *CsNRT1.4A*, *CsNRT1.5B*, *CsNRT1.5C*, *CsNRT1.9*, and *CsNRT1.10*, and downregulated *CsNRT1.5A* and *CsNRT1.8*. LT treatment upregulated the expression level of *CsNRT1.5A*, while exogenous BZR application downregulated the expression level of *NRT1* genes.

**Conclusion:**

These results indicate that exogenous application of EBR alleviated the harmful effects of suboptimal RZT through changes in N metabolism, NH_4_^+^ and NO_3_^−^ flux rates, and *NRT1* gene expression, leading to improved cucumber seedlings growth. Our study provides the first evidence of the role of EBR in the response to suboptimal RZT in cucumber, and can be used to improve vegetable production.

**Electronic supplementary material:**

The online version of this article (10.1186/s12870-019-1838-3) contains supplementary material, which is available to authorized users.

## Background

Environmental factors influence plant growth and developments, and temperature is particularly important. Air temperature is unstable, while root zone temperature (RZT) is considered both stable and more important for study [1]. Temperature and light intensity are key factors affecting plant growth and development [2], which are also influenced by humidity and nutrient availability [3, 4]. Among these factors temperature is very important, that effects overall plant developmental process in short time [3, 7]. The previous studies reported that low temperature stress leads to over production of reactive oxygen species (ROS) and reduce antioxidant enzyme activates, reduction in chlorophylls and photosynthetic capacity, hormonal imbalance, ion uptake and accumulation, thus caused a significant reduction in plant growth and yield [2, 4, 6, 8]. Physiological and molecular studies have shown that plant growth is affected by RZT [3]. RZT influences physiological and biological processes, thereby affecting nutrient uptake and availability [5, 6]. Plant nutrient absorption rate is dependent on RZT [7], and can alter ion balance and nitrogen metabolism [8]; a small increase in RZT could induce large changes in plant growth and development [6].

During winter cultivation, air temperature is unstable, while soil temperature changes slowly and is maintained around 10–15 °C [9]. RZT plays a critical role in plant root physiology, morphology, growth, nutrient and water uptake, and translocation from root to leaf. Even horticultural crops exposed to suboptimal RZT may experience heavy losses of early productivity [3, 5–7]. Earlier studies reported that RZT severely affected nitrogen metabolism in cucumber and reduced growth and yield [2]. These studies demonstrated the importance of RZT on plant growth and development. The potential mechanisms of growth inhibition at ambient RZT may involve water and nutrient uptake rates but are largely unknown.

Nitrogen (N) is an essential macronutrient and its availability in soil affects plant growth and development, as well as all metabolic processes [10]. N is a major constituent of proteins and nucleotides, as well as of chlorophyll, numerous metabolites, and cellular components [11]. Nutrient availability and uptake affect plant growth and development [2]. Ammonium (NH_4_^+^) and nitrate (NO_3_^−^) are the principal soil N sources for plants [12]. Plant fine roots absorb NO_3_^−^ and assimilate NH_4_^+^ into organic N via the GOGAT enzyme, once inside root cells, nitrate (NO_3_^−^) can be reduced to ammonium (NH_4_^+^) by nitrate and nitrite reductase and then assimilated into organic nitrogen through the glutamine synthase (GS)-GOGAT cycle [13–16]. Plants take up nitrate and transport it across the specialized plasma membrane made of root epidermal and cortical cells through a complex transport system [17, 18]. The mechanisms by which nitrate influx and efflux occur have been characterized at both the physiological and molecular levels [13, 19]. Plant cells consist of two nitrate uptake systems; one is a low-affinity system, either constitutive low-affinity system (cLATS) or inducible low affinity transport system (iLATS), which are encoded by *NRT1* genes; the other is a high affinity transport system, either constitutive high affinity transport system (cHATS) or inducible high affinity transport system (iHATS), which are encoded by *NRT2* genes [12, 20]. In *Arabidopsis*, eleven *NRT1* and seven *NRT2* gene homologues have been identified, but a limited number are considered responsible for nitrate uptake from soil [13, 21]. In iHATS, *NRT2.1* and *2.2* and *NRT1.1* in iLATS appear to play a major role in NO_3_^−^ influx [11, 17]. LATS and HATS are involved in root xylem loading and unloading of nitrate (*AtNRT1.5 and AtNRT1.8*) and transport into the leaf [12, 17].

Brassinosteroids (EBRs) are growth-promoting steroid phytohormones in plants [22, 23]. EBRs play vital roles in a wide range of developmental processes in plants from germination to fruit development [24, 25]. Exogenous application of EBRs regulates a variety of physiological, biochemical, and molecular processes which enhance plant tolerance to a variety of abiotic stresses, such as low temperatures, heavy metals, and drought [25, 26]. 24-epibrassinolide (EBR) is the most active synthetic analog of the EBR family and can improve tolerance of low temperatures in pepper, tomato, eggplant, cucumber, and ryegrass [22, 27–30]. The mechanism of EBR activity in plant responses to abiotic stress has been reported [25, 31–33]. EBR promotes plant tolerance to heat, cold, drought, and salinity by correlating with higher expression of stress marker genes, including *heat shock proteins* (*HSPs*) and *cold responsive genes* (*COR*) [25, 29, 34]. EBR Plant exposed to low/cold stress caused negative effects on chlorophyll, photosynthesis, nutrients accumulation and antioxidant enzyme activity, thus leads to reduced plant growth and yield. The pervious study reported that chilling stress downregulate thousands of genes in involved in many developmental process, including chlorophyll and photosynthesis, antioxidant enzymes, hormones and transcriptional factors, while exogenous EBR application reduce the negative effect of chilling/cold stress on pepper seedling [29]. Cold stress caused a significant reduction in antioxidant enzyme activities and increase ROS (reactive oxygen species) accumulation, thus leads to reduce chlorophylls and photosynthetic capacity, while exogenous EBR application reduce the harmful effects and improve growth [37]. These findings are suggested that, EBR enhances biosynthesis of chlorophyll and photosynthetic machinery and activates stress tolerance enzymes, thus reduce the harmful effects of abiotic stresses [35–37]. A previous study reported that EBR regulated nitrogen uptake and metabolism in *Arabidopsis* via the EBR signaling pathway [23, 38]. Additionally, EBR receptor BRI1 (BRASSINOSTEROID INSENSITIVE 1) mutant *bri1–5* induced expression of *AMT1 (*ammonium transporter 1) and *GS* and *GOGAT* encoded genes, showing that EBR signaling transcription factors BES1 (BRI1-EMS SUPPERSSOR 1) and BZR1 (BRASSINAZOLE RESISTANT 1) are involved in pathways of EBR-mediated nitrogen metabolism and uptake [23, 39–41]. A recent study reported that EBR enhanced low temperature and weak light stress tolerance in tomato, by improving nitrogen metabolism, stimulating nitrate and ammonium accumulation, and accelerating nitrogen conversion into free amino acids [28]. These amino acids are involved in biosynthesis of chlorophylls, proteins, primary and secondary metabolites, and enzyme biosynthesis [38]. These findings suggest an active role for EBR in stress and in nitrogen uptake and metabolism to reduce the harmful effect of stress. However, little is known about the role of EBR in nitrate and ammonium ion influx and in regulation of nitrogen metabolism under suboptimal RZT. This study will comprehensively determine the role of EBR in nitrogen uptake, metabolism, and accumulation under suboptimal RZT in cucumber seedlings.

Cucumber, which is widely grown in greenhouses in northern parts of China during summer and winter seasons, is intolerant to suboptimal RZT, leading to large yield losses [2, 9]. Therefore, suboptimal RZT is a major limiting factor for winter cultivation of cucumber in greenhouses [9, 42, 43]. In this study, we investigated the effect of EBR on cucumber seedling physiology and growth under suboptimal RZT. We hypothesized that exogenous EBR applied to leaves may enhance cucumber seedling growth by increasing enzyme activities and expression of genes involved in nitrogen metabolism as well as regulating nutrient uptake (ion influx rate). The key objectives of this study were to: (1) investigate the effect of RZT on plant physiology; (2) examine whether exogenous EBR application can effectively enhance nitrogen metabolism and uptake rate (ion influx rate); and (3) examine whether exogenous EBR regulates NRT1 expression in cucumber. The results could improve understanding of the role of EBR in nitrogen metabolism, uptake, and response to RZT, which is useful for greenhouse vegetable production.

## Results

### Effect of EBR on cucumber seedlings growth under suboptimal RZT

Cucumber seedlings growth and growth-related parameters were investigated seven days after exposure to various treatments of suboptimal RZT and EBR, as presented in Table 1. Suboptimal RZT significantly reduced the growth of cucumber seedlings (Table 1**)**. Briefly, plant height, hypocotyl dimeter, leaf area, total fresh weight and seedling index were decreased by 26.59, 29.43, 28.71, 38.04 and 39.18% respectively, in the LT treatment compared to the NT treatment, but decreased by 31.40, 27.54, 32.75, 38.19, and 33.89% respectively, when compared to EBR treated seedlings. The differences between NT and EBR treatments were not significant. Moreover, the plant height, root fresh weight, shoot fresh weight, and total fresh weight of cucumber seedlings in the EBR treatment were significantly higher than in the LT and BZR treatments. These results suggest that EBR alleviated the harmful effects of suboptimal RZT temperature, thus leading to improved cucumber seedlings growth.

**Table 1:** Changes in cucumber seedling growth after seven days under suboptimal RZT with EBR and BZR application

Treatments	Plant Height (cm)	Hypocotyl Diameter (mm)	Leaf Area (mm^2^)	Total FW (g)	Seedling Index
NT	6.13 ± 0.41 a	3.84 ± 0.07 a	678.33 ± 37.78a	3.97 ± 0.15 a	3.88 ± 0.36 a
LT	4.50 ± 0.35 b	2.71 ± 0.08 b	483.55 ± 50.11 b	2.46 ± 0.29 b	2.36 ± 0.41 b
EBR	6.56 ± 0.34 a	3.74 ± 0.08 a	719.01 ± 33.86 a	3.98 ± 0.27 a	3.57 ± 0.31 a
BZR	4.39 ± 0.42 b	2.96 ± 0.17 b	440.27 ± 32.83 b	2.29 ± 0.12 b	2.23 ± 0.38 b

Data are the means of four replicates with standard deviation (±SD). Means followed by the same lowercase letter are not significantly different by the least significant difference (LSD) test at *P* = 0.05

### Effect of exogenous EBR application on endogenous accumulation of EBR

**Fig. 1:**
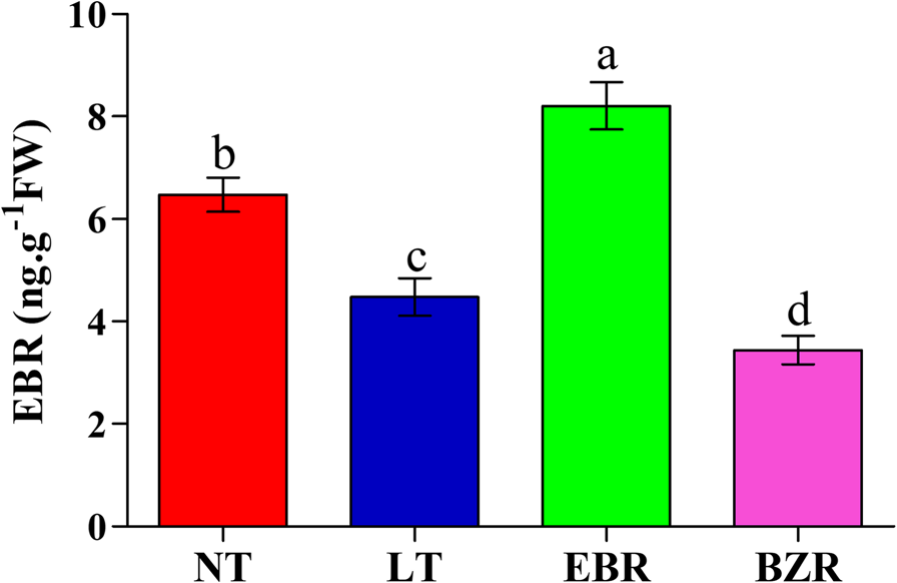
Changes in endogenous EBR contents in leaves of cucumber seedlings after suboptimal RZT with EBR and BZR application. Leaf samples were harvested seven days after treatment. Data are the means of four replicates with standard deviation (SD) shown by vertical bars on top of columns. Columns with the same lowercase letter are not significantly different by the least significant difference (LSD) test at *P* = 0.05

The endogenous EBR contents were measured in cucumber leaves seven days after exposure to suboptimal RZT. As shown in Fig. 1, the LT (control; suboptimal RZT) treatment significantly decreased endogenous EBR accumulation in the leaf by 28.24% compared with the control (NT, normal RZT). Moreover, exogenous EBR application significantly increased endogenous EBR accumulation in cucumber leaf by 43.34 and 58.09% compared with LT and BZR treatments, respectively, under suboptimal RZT, while it increased by 26.66% over that of the NT treatment (normal RZT).

### Effect of EBR on root activity and bleeding rate

**Fig. 2:**
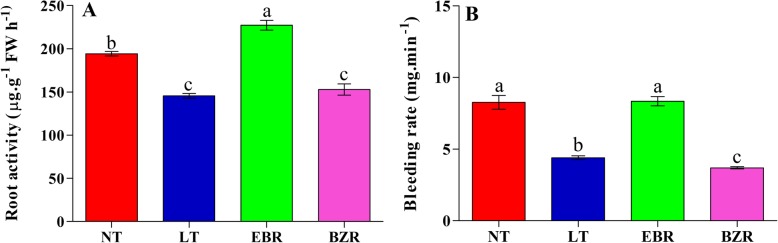
Changes in root activity (A) and bleeding rate (B) cucumber seedling after seven days under suboptimal RZT with EBR and BZR application. Treatments with the same lowercase letter are not significantly different by the least significant difference (LSD) test at *P* = 0.05

Root activity is an important parameter for suboptimal RZT because it reflects the strength of overall metabolic processes in root tissues including respiration, oxidation, and enzymatic activities involved in nutrient uptake. Suboptimal RZT caused a significant reduction in root activity of cucumber seedlings, but these increased significantly in the EBR treatments (Fig. 2 A. Briefly, the EBR treatment increased root activity by 17.05, 35.98, and 32.76%, when compared with the NT, LT, and BZR treatments, respectively. Additionally, the LT and BZR treatments negatively affected the root activity of cucumber seedlings, with reductions of 25.07 and 21.29%, respectively, compared to the NT treatment.

Suboptimal RZT also negatively affected bleeding rate (Fig. 2 B. In the LT treatment, bleeding rate was reduced by 46.73% compared to that of the NT treatment, while exogenous EBR application significantly increased bleeding rate by 47.31, and 55.65%, compared to that of the LT and BZR treatments (Fig. 2 B). The bleeding rate did not differ detectably between the NT and EBR treatments. These findings suggest that suboptimal RZT negatively affected root activity and bleeding rate and caused a significant reduction in growth.

### Effect of EBR on NR, NiR, GS and GOGAT enzyme activities

**Fig. 3:**
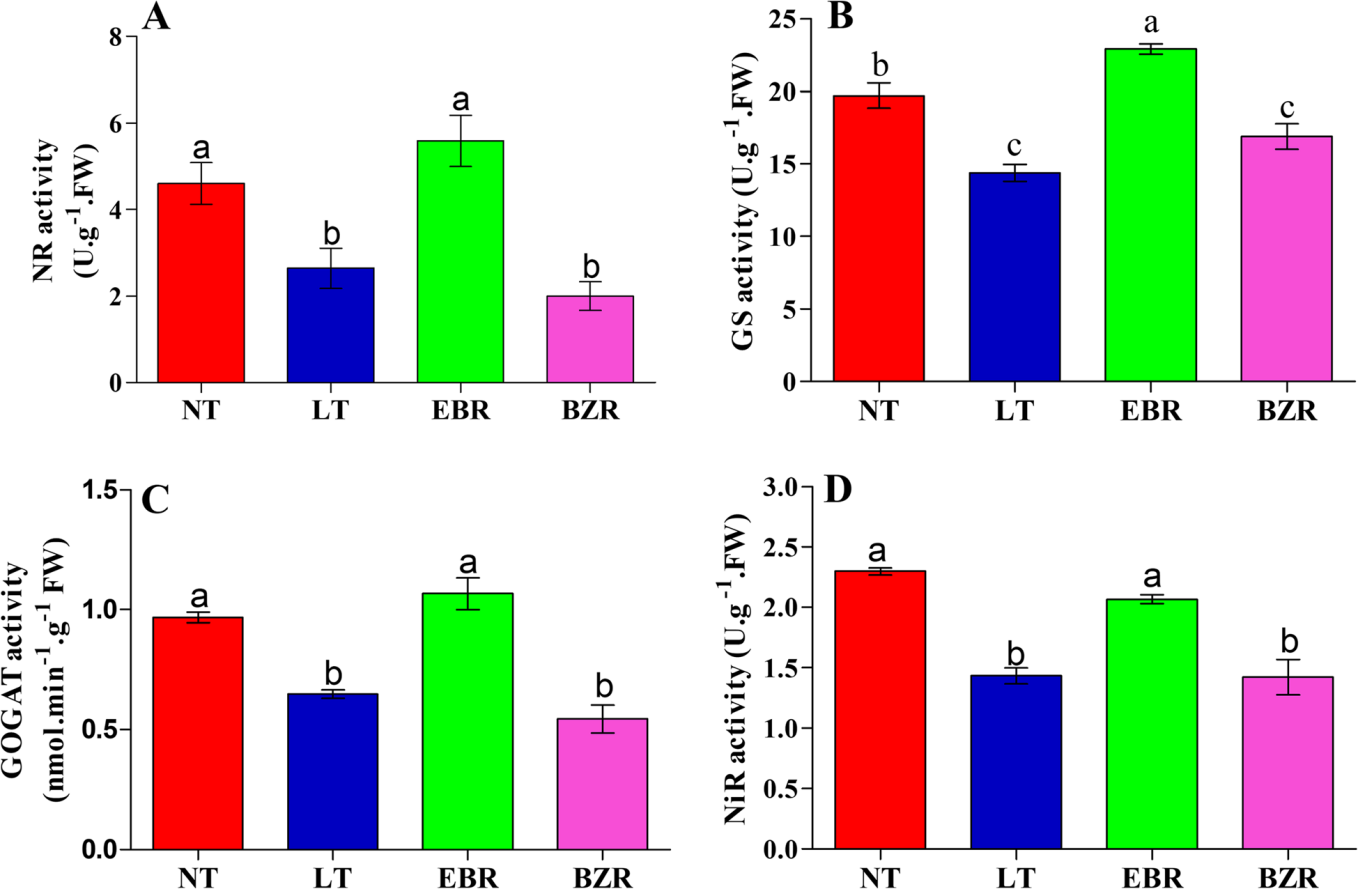
Effect of EBR on A; nitrate reductase (NR), B; glutamine synthetase (GS), C; glutamate synthase (GOGAT), and D; nitrite reductase (NiR) activities in leaves of cucumber seedlings under suboptimal RTZ. Treatments with the same lowercase letter are not significantly different by the least significant difference (LSD) test at *P* = 0.05

Nitrate reductase (NR), nitrite reductase (NiR), glutamine synthetase (GS), and glutamate synthase (GOGAT) are key enzymes involved in nitrogen metabolism. We investigated these enzymes in leaves of cucumber seedlings after seven days of suboptimal RZT. The enzymes involved in nitrogen metabolism (NR, NiR, GS, and GOGAT) were adversely affected by suboptimal RZT (LT), when compared to the NT and EBR treatments, as presented in Fig. 3. Exogenous EBR significantly increased the activities of NR, NiR, GS, and GOGAT under suboptimal RZT, as compared to the LT treatments, but differences between the NT and EBR treatments were not significant (Fig. 3). Glutamine synthetase activity was significantly higher in the EBR treatment compared to the NT treatment (Fig. 3 B). These findings suggested that EBR regulates the activities of NR, NiR, GS, and GOGAT under suboptimal RZT in cucumber seedlings.

### Effect of EBR on NH_4_^+^ and NO_3_^−^ fluxes rate

**Fig. 4:**
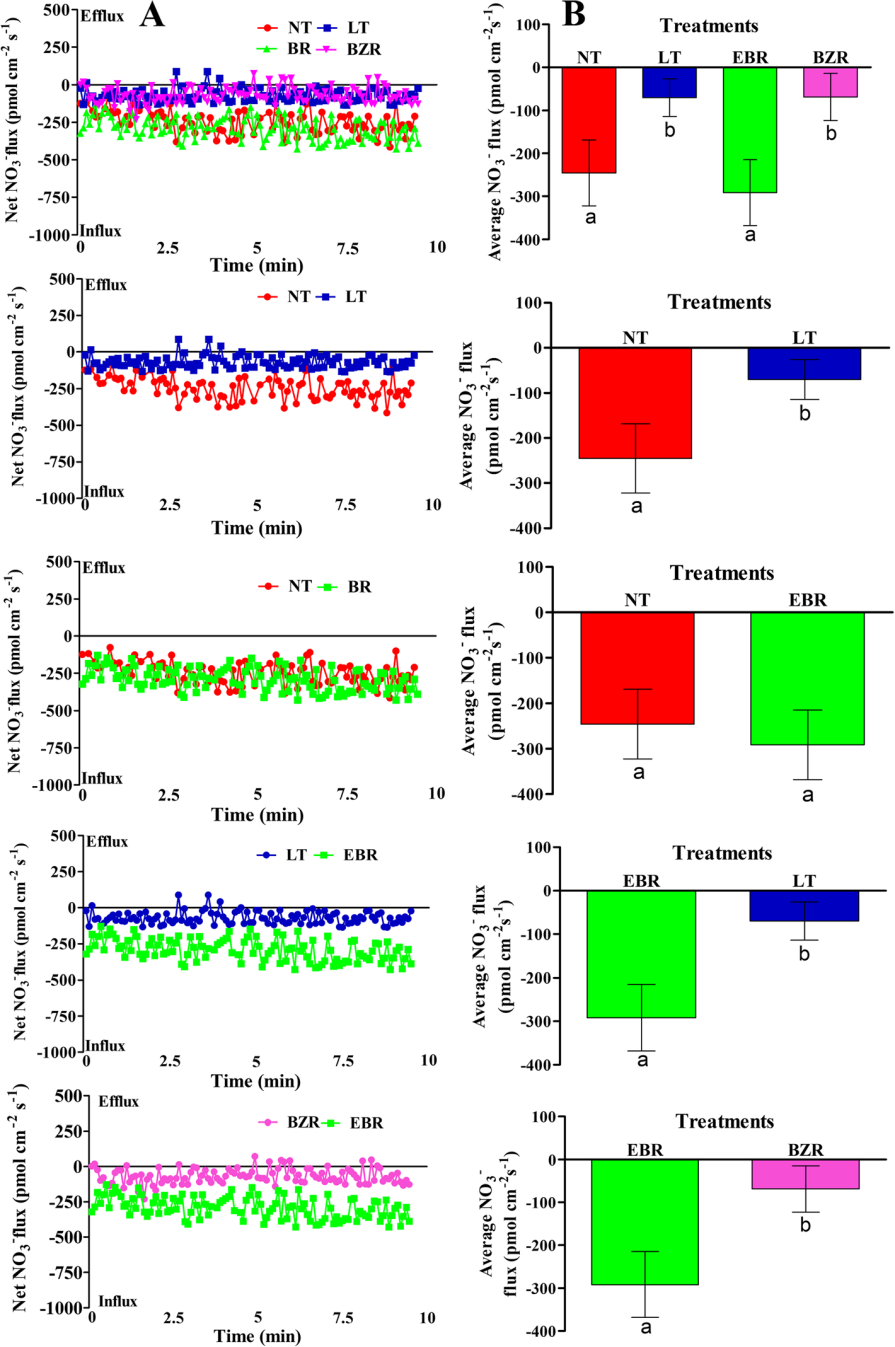
Effects of suboptimal RZT and EBR on NO_3_^−^ (A; scatter NO_3_^−^ flux rate, B; average NO_3_^−^ flux rate) flux rate in roots of cucumber seedlings under suboptimal RZT treatment. Flux rate was recorded for 10 min in roots seven days after treatment. Each point is the mean of nine individual seedlings and bars indicate standard deviations. Treatments with the same letters are not significantly different by the least significant difference (LSD) test at *P* = 0.05

**Fig. 5:**
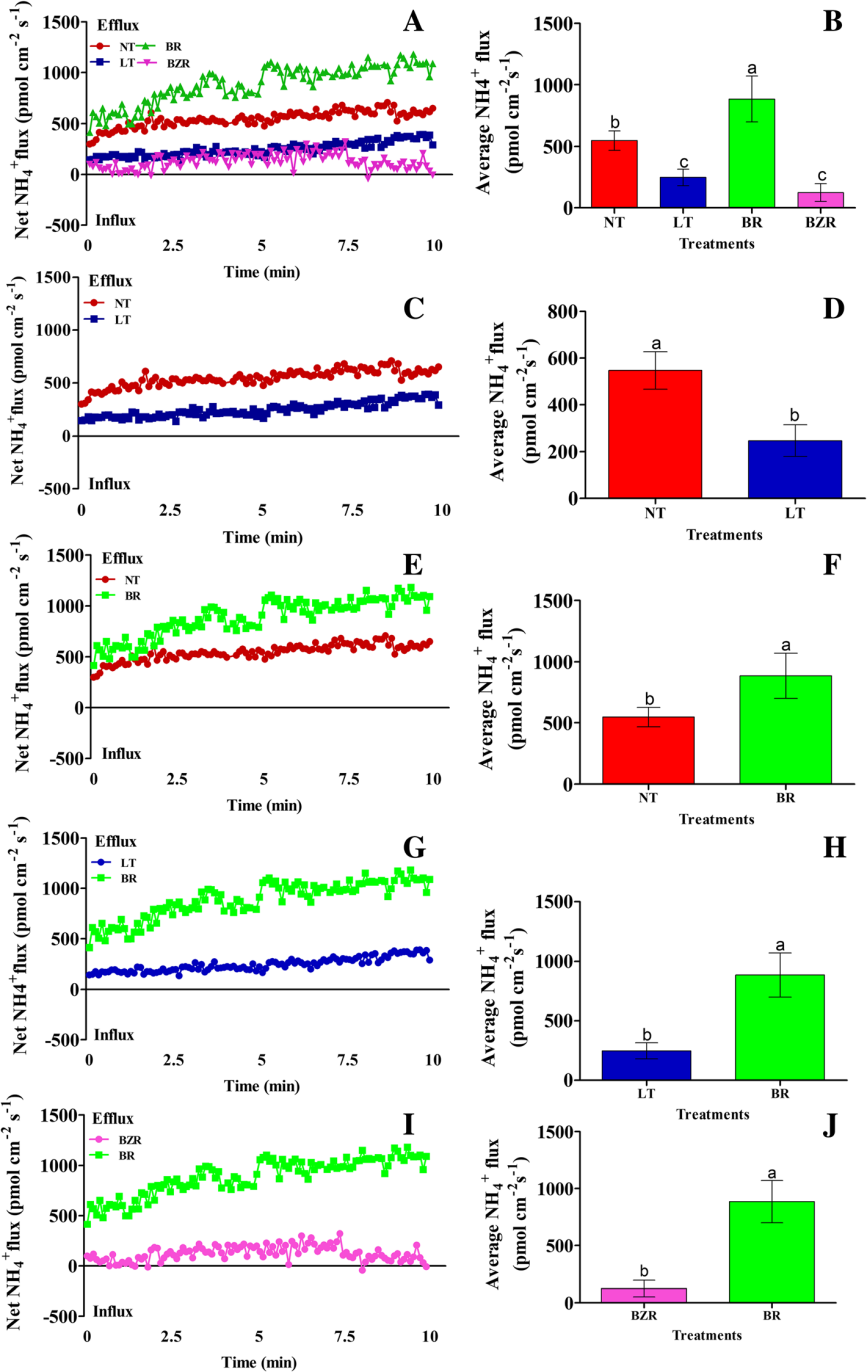
Effects of suboptimal RZT and EBR on NH_4_^+^ (A; scatter NH_4_^+^ flux rate, B; average NH_4_^+^ flux rate) flux rate in roots of cucumber seedlings under suboptimal RZT treatment. Flux rate was recorded for 10 min in roots seven days after treatment. Each point is the mean of nine individual seedlings and bars indicate standard deviations. Treatments with the same letters are not significantly different by the least significant difference (LSD) test at *P* = 0.05

The NH_4_^+^ and NO_3_^−^ flux rates were investigated in the roots of cucumber seedlings seven days after exposure to suboptimal RZT. Suboptimal RZT negatively affected NH_4_^+^ and NO_3_^−^ flux rates, but exogenous EBR application significantly increased NO_3_^−^ and NH_4_^+^ flux rates in root of cucumber seedlings (Figs. 4 & 5).

The results indicated that, NO_3_^−^ influx rate in cucumber roots were also adversely affected by suboptimal RZT, as shown in Fig. 4. The average influx rates indicate that exogenous EBR application significantly increased NO_3_^−^ influx rate by 72.11 and 86.02%, when compared with LT and BZR treatments, respectively (Fig. 4). NO_3_^−^ flux rate decreased significantly (by 54.85%) in the LT compared to the NT treatment. Additionally, EBR increased the nitrate influx rate by 15.75% compared to that of the NT treatment, but this difference was not significant (Fig. 4). The influx rate of nitrate did not differ significantly between LT and BZR (Fig. 4**)**.

Similarly, the NH_4_^+^ efflux rate was significantly higher in the EBR treatment, but significantly lower in the BZR and LT treatments (Fig. 5). Exogenous EBR application increased average NH_4_^+^ efflux rate by 15.75, 71.01, and 76.44%, as compared to the NT, LT, and BZR treatments, respectively (Fig. 5). The NT treatment showed a 71.53% increase in average NH_4_^+^ flux rate compared to the LT treatment (Fig. 5). The difference between the NT and EBR treatments was not significant from 0 to 2.5 min (Fig. 5) but became significant over time (Fig. 5). The differences between the LT and BZR treatments were not significant (**Fig. 5**). These results suggest that suboptimal RZT caused negative effects on cucumber roots and led to a reduction in the flux rate of NO_3_^−^ and NH_4_^+^ which decreased cucumber seedling growth rate. Additionally, exogenous EBR application reduce the detrimental effects of suboptimal RZT through increasing NH_4_^+^ and NO_3_^−^ flux rates.

### The effect of EBR on N accumulation under suboptimal RZT

**Fig. 6:**
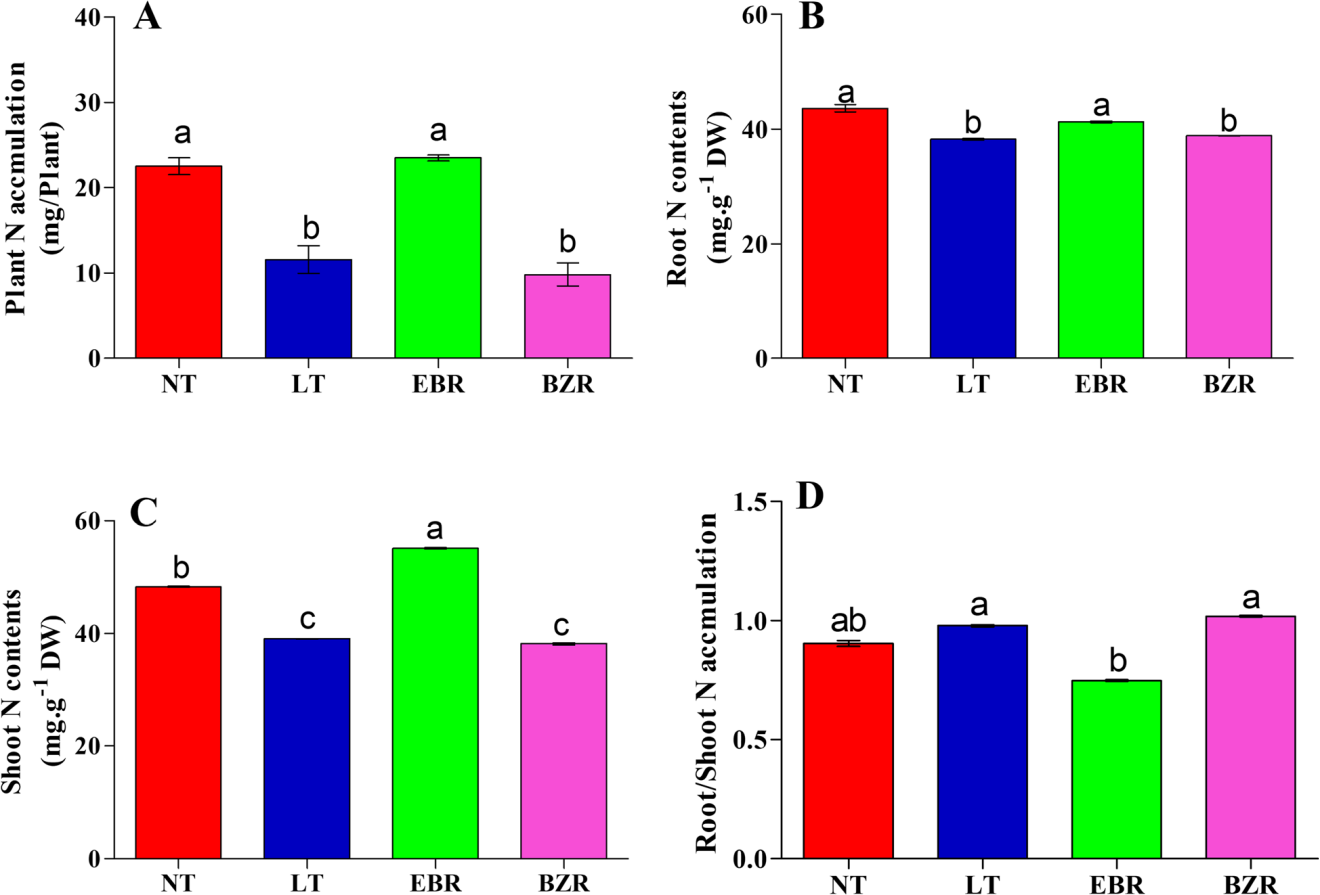
Nitrogen accumulation in cucumber (root and shoot) seven days after exposure of suboptimal RZT Treatments with the same letters are not significantly different by the least significant difference (LSD) test at *P* = 0.05

Nitrogen (N) is a major component of proteins, amino acids, nucleic acids, chlorophyll, and enzymes, and thus its accumulation is important for plant growth and development. The results of present study indicated that the LT treatment caused a severe reduction in N accumulation in cucumber root, leaf, and shoot tissues when exposed to suboptimal RZT for seven days, with decreases of 19.30, 12.41 and 48.56%, respectively, when compared with those tissues under the NT treatment (Fig. 6). Further, exogenous EBR application significantly increased the root and shoot total N contents, and plant N accumulation, by 7.41, 29.27, and 50.68%, respectively, when compared with those values for the LT treatment. The maximum root/shoot N accumulation was reported in LT and BZR treated seedlings over that observed in the NT and EBR treatments (Fig. 6**).**

### Effect of EBR on NO_3_^−^ and NH_4_^+^ contents

**Fig. 7:**
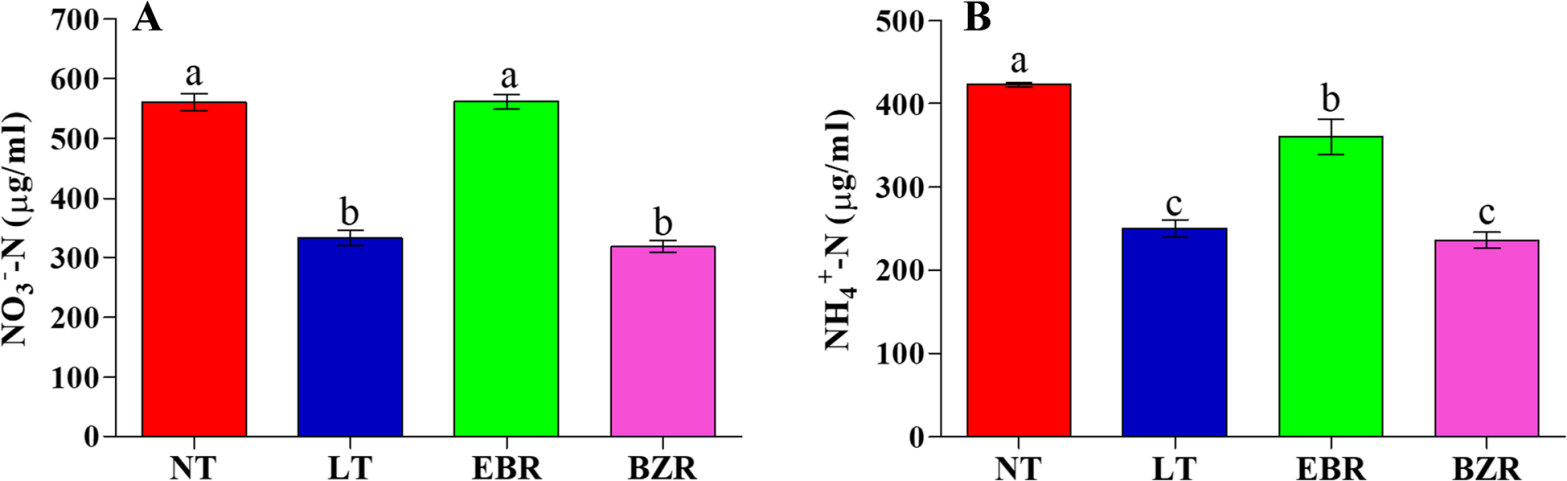
EBR regulates NO_3_^−^-N and NH_4_^+^-N contents in cucumber leaves under suboptimal RZT. Treatments with the same lowercase letter are not significantly different by the least significant difference (LSD) test at *P* = 0.05

Suboptimal RZT (LT) significantly decreased the accumulation of nitrate and ammonium in leaves of cucumber seedlings **(**Fig. 7). Moreover, exogenous EBR application significantly increased nitrate and ammonium accumulation upon exposure to suboptimal RZT by 40.61 and 30.28%, respectively, compared to the LT treatment, and by 43.32 and 34.44%, compared with the BZR treatment. The NT treatment significantly increased nitrate and ammonium contents by 48.77 and 40.66%, respectively, compared to those of the LT treatment. Additionally, nitrate content in the EBR and NT treatments were not significantly different (Fig. 7 A), but ammonium contents were significantly higher (14.89%) in NT when compared to EBR (Fig. 7 B).

### Nitrate transporter (NRT1) gene expression

**Fig. 8:**
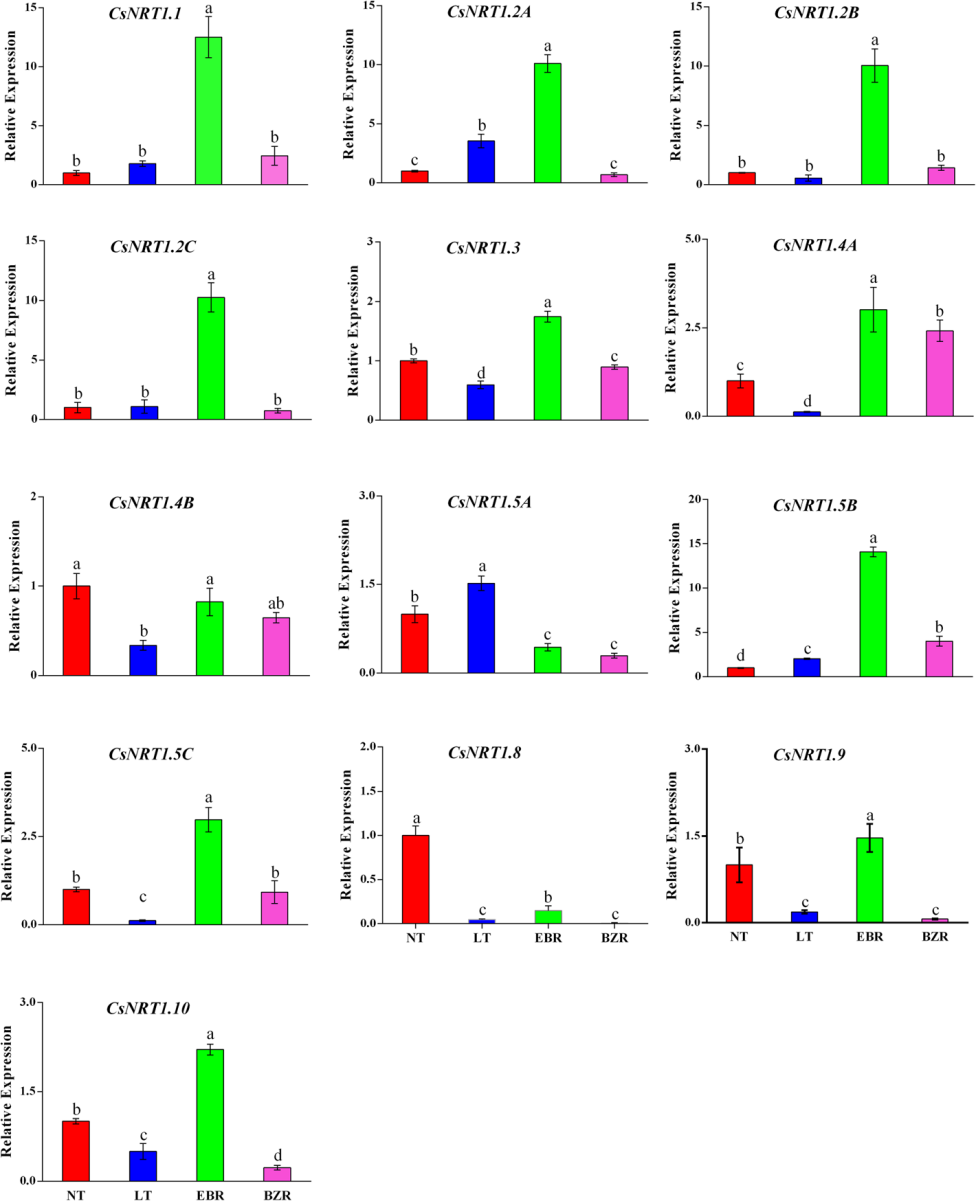
EBR regulates transcript levels of NRT1 genes in leaves of cucumber seedlings under suboptimal RZT. Leaf samples were harvested seven days after treatment. Columns indicate the means of independent measurements of four replications per treatment and bars indicate the SD of the mean. Treatments with the same lowercase letter(s) are not significantly different by the least significant difference (LSD) test at P = 0.05

The effects of suboptimal RZT on nitrate transporter 1 (*NRT1*) genes (low affinity transporters) were investigated in cucumber leaves under suboptimal RZT (Fig. 8). LT significantly downregulated the transcription levels of *NRT1* genes, but these genes were significantly upregulated by exogenous EBR application under suboptimal RZT.

Transcription levels of *CsNRT1.1, CsNRT1.2A, CsNRT1.2B, CsNRT1.2C, CsNRT1.3, CsNRT1.4A, CsNRT1.5B, CsNRT1.5C, CsNRT1.9*, and *CsNRT1.10* significantly increased in EBR treated seedlings under suboptimal RZT. Additionally, EBR downregulated the expression of *CsNRT1.5A* and *CsNRT1.8*, while *CsNRT1.4B* showed the same trend in the NT, EBR, and BZR treatments, but was downregulated by the LT treatment. Among the LATS gene family, *CsNRT1.1, CsNRT1.2A, CsNRT1.2B, CsNRT1.2C*, and *CsNRT1.5B* showed higher expression than did other members of this family when treated with exogenous EBR under suboptimal RZT. These findings indicated that EBR activated the expression of *NRT1* genes and led to increased N metabolism, thus improving cucumber seedling growth under suboptimal RZT.

## Discussion

EBR is a growth-promoting steroid hormone which plays an active role in a wide range of developmental processes, including abiotic stress tolerance [25, 34, 35]. EBR increases tolerance to abiotic stresses including chilling [29], heat [44], drought [10], and salinity [45]. The previous study shows that EBR regulate thousands of genes in pepper to reduce the harmful effects of chilling stress [46]. Nitrogen is an essential, that’s promotes plant growth and development, as well as alleviate the inhibitory effects of abiotic stresses, but their metabolism is sensitive suboptimal RZT [2]. The previous studies suggested that nitrogen metabolism plays a fundamental role in biosynthesis of chlorophyll and photosynthetic capacity [8]. It has been widely observed that abiotic stress induced reduction in chlorophyll and photosynthetic capacity accompanied by the decrease in the nitrogen metabolic enzyme activities, like NR, NiR, GS, GOGAT [2, 6, 12]. Exogenous EBR application alleviates the harmful effects of low temperature and weak light stress through enhancing the nitrogen metabolism and photochemical efficiency in tomato seedling [28]. Suboptimal RZT causes a significant reduction in plant growth and growth-related parameters [1, 2]. Previous studies reported that exogenous EBR application increased low temperature stress tolerance, regulated levels of endogenous plant hormones (including EBR contents), and regulated expression of an EBR biosynthesis gene (*CsDWF*) [34, 47, 48]. In the present study, endogenous EBR contents increased in EBR treated seedlings (Fig. 1) and resulted in significant increases in cucumber seedling growth under suboptimal RZT (Table 1). These findings are in line with those of a previous study, who reported that exogenous EBR increased cucumber and pepper seedlings growth under low temperature stress [29, 48].

Suboptimal RZT negatively affects plant growth by affecting chlorophyll, photosynthetic capacity and nitrogen metabolism [2, 6]. Earlier studies reported that suboptimal RZT severely affected plant and root physiology through changes to ion balance [6], nitrogen metabolism [49], chlorophyll [50], photosynthesis [4, 7, 51], and antioxidant enzyme activities and that negatively affected plant metabolic processes [2, 42, 50, 52], thus leading to reduced plant growth (Table 1). Regulation of RZT leads to instantaneous changes in root physiology and affects root metabolism and morphology [4, 6], thereby impacting critical root functions such as nutrient uptake and absorption rate [42]. Additionally, roots are the main source of water and minerals uptake and are responsible for translocation from the soil to plant tissues [49, 53]. A previous study suggested that low root zone temperature caused a significant reduction in root activity, thus leading to a significant reduction in growth [54]. Root activity reflects the strength of all metabolic processes in the root system, including respiration, oxidation, and enzyme activities, and nutrient absorption and translocation from roots to shoots, which are closely related to all developmental processes [55]. Our results demonstrated that, LT treatment severely reduced root activity and bleeding rate, while these significantly increased in cucumber seedlings treated with exogenous EBR (Fig. 2). The correlation analysis suggests the positive correlation between root activity, bleeding rate with root ion flux, as presented in Table 2. These findings suggest that EBR plays an important role in alleviating the harmful effects of suboptimal RZT by regulating root activity and bleeding rate in cucumber seedlings. The results are consistent with those of earlier studies, which found that RZT significantly affected plant biomass, bleeding rate, and root activities [1, 55]. Moreover, suboptimal RZT both increases root oxygen demand and decreases oxygen concentration available in soil, which can result in hypoxic conditions and reduce root and shoot growth [56].

**Table 2:** Correlation (Pearson) analysis between root activity, bleeding and influx rate

Treatment	Root Activity	Bleeding rate	NO_3_^−^ flux rate	NH_4_^+^ flux rate
Root Activity	1	0.9213	−0.9763	0.9725
Bleeding rate	0.9213	1	−0.9830	0.9183
NO_3_^−^ flux rate	−0.9763	−0.9830	1	−0.9560
NH_4_^+^ flux rate	0.9725	0.9183	−0.9560	1

The level of significance was *P* = 0.05

Nitrogen is an important constituent of basic nitrogen-containing compounds such as amino acids, proteins, chlorophylls, and nucleotides, which play important roles in plant growth and development [12, 17, 57, 51]. Therefore, understanding the physiological and molecular mechanisms of nitrogen metabolism and responses to suboptimal RZT is important for agronomic approaches to enhance nitrogen use efficiency in crops and reduce losses [13, 20, 58]. Most plants absorb inorganic nitrogen from soil as ammonium (NH_4_^+^) and nitrate (NO_3_^−^), which serve as N source [59]. NH_4_^+^ can be assimilated to glutamine by the GS and GOGAT enzymes [59, 60]. Plant roots absorb NO_3_^−^ which is then converted into NH_4_^+^ by NR and NiR enzymes for synthesis of amino acids, proteins, and nucleotides [17], thus N accumulation in roots and shoots is important for plant growth and development [58]. A previous study revealed that RZT significantly reduced N accumulation in leaf and root tissues of cucumber, and suggested that N uptake depends on the temperature root zone [42]. Our results indicated that N contents (total N, nitrate, and ammonium contents) under suboptimal RZT were much lower than those of EBR treated seedlings, are suggested that EBR can reduce the harmful effects of suboptimal RZT, as presented in Fig. 6. Under suboptimal RZT, root activity and bleeding rate (Fig. 2) were significantly lower than in the EBR treatments, which indicated that EBR alleviates the harmful effects induced by suboptimal RZT and may explain why EBR increased N accumulation (Fig. 6). These findings are suggested that, suboptimal RZT reduce N accumulation, thus leads to reduce cucumber seedlings growth (Table 1). The results build upon those of previous studies in which RZT negatively affected plant growth through reduced nutrient accumulation [2, 6, 61].

Enzyme activities are very sensitive and reduce very quickly under abiotic stresses [42, 49, 52, 62]. Previous studies indicated that exogenous EBR application positively regulated the activities of enzymes involved in nitrogen metabolism (NR, NiR, GS, and GOGAT) [28, 63]. We investigated the activity of these enzymes involved in N metabolism, as presented in Fig. 3. Our results indicated that the activities of these enzymes (NR, NiR, GS and GOGAT) under suboptimal RZT were much lower than in the EBR treatment. We proposed that suboptimal RZT might have cause a reduction in enzyme activities (Fig. 3), thus leading to a significant reduction in nitrate and ammonium contents (Fig. 7). Our results suggested that the NR, NiR, GS and GOGAT enzymes activities, and assimilation of nitrate and ammonium were promoted after EBR application in cucumber, as exposed to stress. These findings are supported by an earlier study which reported that EBR enhanced the activity and expression levels of GS and GOGAT enzymes and genes in *Arabidopsis* and concluded that BZR1 and BES1 transcription factors might be involved in different pathways of BR-mediated nitrogen metabolism and uptake [14, 23, 52]. Therefore, exogenous EBR application regulated N metabolism under suboptimal RZT, thus leading to improved growth, as presented in Table 1.

Suboptimal RZT significantly reduced root activity and bleeding rate, both of which may affect nutrient and water uptakes in cucumber seedlings (Fig. 2). N acquisition in plants is primarily regulated by plant hormones [28, 64], which may activate nitrogen signaling pathway to promotes the flux rate of NH_4_^+^ and NO_3_^−^ ion in roots [65]. Previous studies reported that ion flux is sensitive to external stimuli (abiotic stresses), which can cause a significant reduction in ion uptake/flux rate [1, 2]. Our previous results suggested, that suboptimal RZT severely reduced the enzyme activities involved in N metabolism (Fig. 3). Therefore, we speculated that EBR plays a role in NH_4_^+^ and NO_3_^−^ flux rates in cucumber roots under suboptimal RZT. As predicted, NH_4_^+^ efflux and NO_3_^−^ influx rate in cucumber roots under suboptimal RZT were significantly lower than in the EBR treatments (Figs. 4 & 5). Additionally, we compared NH_4_^+^ efflux and NO_3_^−^ influx rates under the EBR treatment with those of the NT and BZR treatments to make clear the role of EBR. Our findings suggested that EBR increased NH_4_^+^ and NO_3_^−^ flux rates and reduced harmful effects, thus leads to significant increment in nitrogen accumulation (Fig. 6). The earlier studies suggested that ammonium and nitrate flux rates are affected by abiotic stresses [8, 46, 66]. EBR is a steroid hormone and induces plant tolerance to a variety of stresses [51, 64, 67, 68]. These findings are suggested that, EBR reduces the negative effect of suboptimal RZT, through increasing NH_4_^+^ and NO_3_^−^ flux rates under suboptimal RZT [28, 69, 70]. Additionally, *NRT1s* protein family plays an important role in nitrate absorption from soil and translocation to various plant tissues, and these proteins were significantly upregulated by exogenous EBR application, as presented in Fig. 8. Activation of the EBR signal transduction pathway may lead to upregulation of the *AMT1*, *NRT1,* and *GS/GOGAT* genes in *Arabidopsis* [23, 71, 72]. The positive correlation was reported between ion flux rate, root activity and bleeding rate (Table 2), are suggesting that EBR minimized the detrimental effects induced by suboptimal RZT, and could explain mechanism of NH_4_^+^ and NO_3_^−^ influx rates, that’s significantly higher in EBR treated seedlings. These findings provide evidence that EBR enhanced NH_4_^+^ and NO_3_^−^ acquisition capacity, which may have significantly increased nitrogen metabolism and cucumber seedling growth (Table 1).

Plants absorb nitrate and ammonium from soil through various transporters: NO_3_^−^ is absorbed by *NRT1* protein family members and incorporated into amino acids through the GS and GOGAT enzymes [2, 15, 73]. The *NRT1s* gene family is responsible for the overall mechanism of nitrate absorption and translocation in plants [12, 74]. However, suboptimal RZT is unfavorable for N acquisition and metabolism, which significantly reduces horticultural production [2, 7, 54]. We investigated the effect of EBR using transcript levels of *NRT1* genes, which play specific roles in nitrate absorption and translocation in various plant tissue. Previous studies reported a positive correlation between the flux rates of nitrate and ammonium with the transcript levels of *NRT1* genes [13]. The results of this study indicated that exogenous EBR application significantly induced the expression of *CsNRT1* genes, which were downregulated by suboptimal RZT and exogenous BZR (Fig. 8). These findings suggest that exogenous EBR activated the expression levels of *NRT1* genes, potentially contributing to the observed increase in nitrate (Fig. 4) and ammonium flux rates (Fig. 5), enzyme activities (Fig. 3), and N accumulation in leaf and root tissues of EBR treated seedlings (Fig. 6). The *CsNRT1s* gene upregulated after exogenous EBR application, are suggested that BZR1 and BES1 transcription factors might be involved directly in *NRT1s* regulation [14, 15, 23]. A previous study reported that *CsNRT1* genes showed variable expression patterns across plant tissues and suggested that *NRT1*s proteins are primarily responsible for nitrate absorption and translocation. Among these, *CsNRT1.1, CsNRT1.3, CsNRT1.4B, CsNRT1.5A* and *CsNRT1.8* regulate nitrate, whereas other members of this family (*CsNRT1.9, CsNRT1.2 s, CsNRT1.4A, CsNRT1.5B,* and *CsNRT1.5C*) also appear to play distinct physiological roles in plants [12, 16]. These variable roles help to explain why *NTR1* genes showed different expression patterns, as presented in Fig. 8. The EBR signaling pathway is known to mediate *AMT1* encoded genes and induce N-metabolism and uptake in *Arabidopsis* [23]. In our study, similar temporal levels of *NRT1* genes were observed, indicating that suboptimal RZT down-regulated *NRT1* genes and significantly reduced growth [2, 7]. In a recent study, EBR affected N metabolism by increasing nitrate and ammonium contents and enzyme activities (NR, NiR, GS, and GOGAT), which increased the tolerance of tomato to low light and temperature [23, 28, 49]. These findings are concluding that, exogenous EBR alleviates the adverse effect of suboptimal RZT by modulating nitrogen metabolism, thus leading to improved cucumber seedling growth.

## Conclusion

**Fig. 9:**
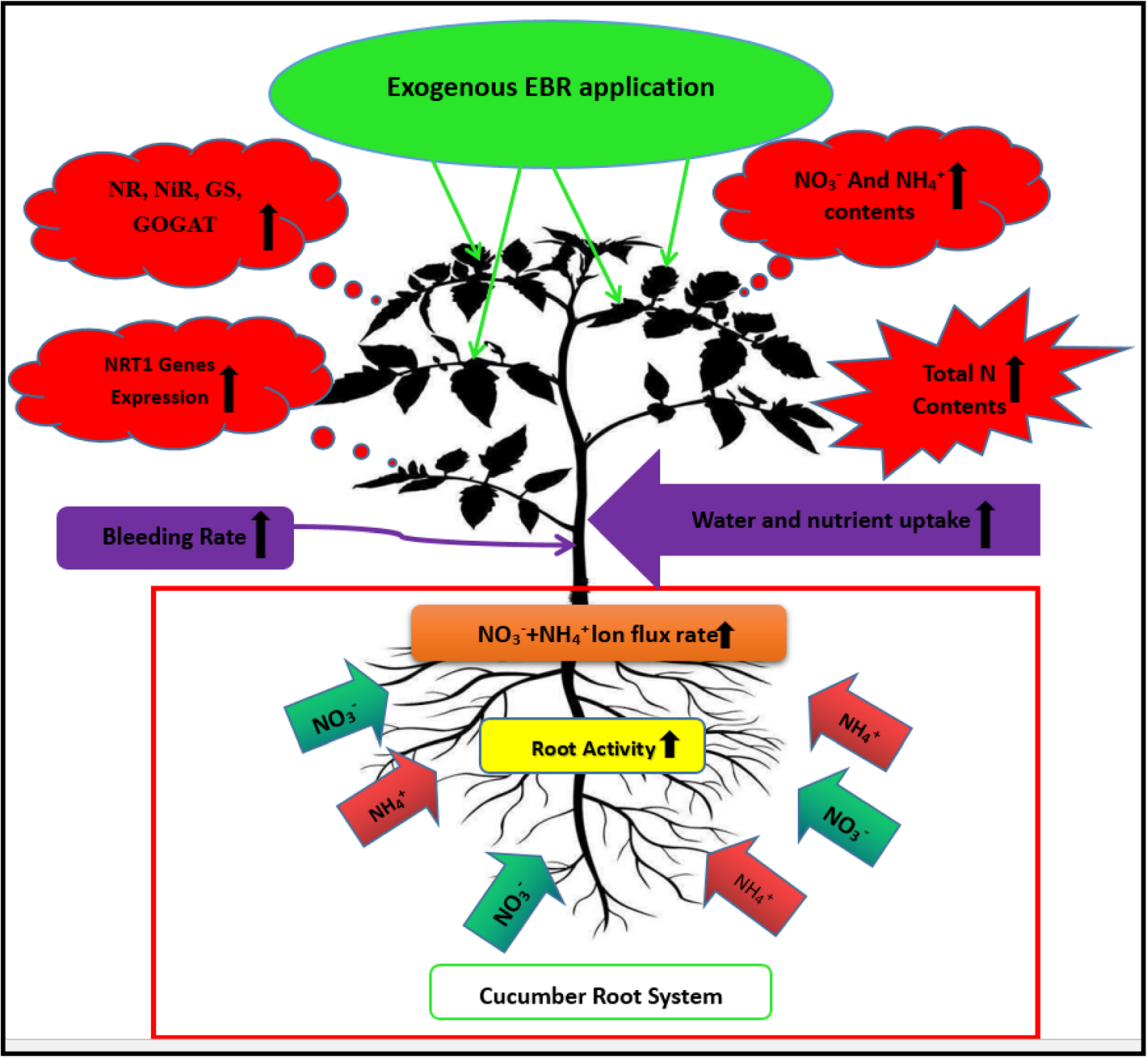
The regulatory mechanism of EBR to alleviate the deleterious effect of suboptimal RZT

In summary, suboptimal RZT caused harmful effects on cucumber seedlings and severely reduced plant growth, while exogenous EBR application reduced these detrimental effects of suboptimal RZT. Exogenous EBR application could effectively regulate nitrogen metabolism via increasing activity of enzymes involved in nitrogen metabolism (NR, NiR, GS, and GOGAT), and transcript levels of *NRT1* genes, nitrate and ammonium influx rate, which might have a positive effect on growth of cucumber seedlings exposed to suboptimal RZT, as presented in Fig. 9. Our study provides the first evidence of the role of exogenous EBR in transcriptional regulation of *NRT1* genes, NO_3_^−^ and HN_4_^+^ ion flux rates under suboptimal RZT in cucumber. This study provides new insights into EBR signal transduction pathway and interactions with *NRT1* family genes and responses under suboptimal RZT. Future studies will need to focus on the molecular mechanism of the interaction between EBR and *NRT1* genes during signal transduction in plants.

## Methods

### Plant material and growth conditions

Cucumber (*Cucumis sativus* L. Cv. Zhongnong 26) seeds were obtained from; The Institute of Vegetables and Flowers, Chinese Academy of Agricultural Sciences, Beijing, China. The detail method and treatments are same as previously described by Anwar et al. [75]

### Measurement of growth

Plant height and root length were determined using a ruler, while fresh weight was determined using a digital balance [9]. The seedling index was used to calculate as (stem thickness / plant height + root FW / shoot FW) * FW of whole plant.

### Determination of root activity and bleeding rate

The root activity and bleeding rate were investigated six days after exposure to suboptimal RZT. Eight seedlings per treatment were cut below the cotyledons and the incisions were quickly covered with absorbent cotton to collect bleeding sap for two hours. The bleeding rate was calculated from the weight increments of absorbent cotton after two hours. The root activity was determined using TTC (C_19_H_15_CIN_4_) [76].

### Enzyme assays

Enzyme activities of nitrate reductase (NR), nitrite reductase (NiR), glutamine synthetase (GS), and glutamate synthase (GOGAT) were determined using assay kits (COMINBIO) with a UV-1800 spectrophotometer following the manufacturer’s instructions [77].

### Determination of nitrate and ammonium contents

Nitrate (NO_3_^−^) and ammonium (NH_4_^+^) contents in the leaf were determined after seven days of exposure to suboptimal RZT, using the methods described by [28]. The OD was read at 410 nm and 625 nm, and contents were calculated using a standard curve [28].

### Measurement of NO_3_^−^ and NH_4_^+^ flux rate at the root surface with NMT

Net NH_4_^+^ flux of cucumber seedlings roots was measured by using Non-invasive Micro-test Technology (NMT Physiolyzer®, Younger USA LLC, Amherst, MA 01002, USA) in Xuyue (Beijing) Sci. & Tech. Co., Ltd., Beijing, China [78].

The cucumber roots were fixed to the bottom of petri dish using resin blocks and filter paper strips, the root tip was exposed, then incubated in the testing solution (2.625 mM Ca (NO_3_)_2_, 0.1 mM MgSO_4_, 0.25 mM NH_4_NO_3_, 0.3 mM MES, pH 6.0) for 20 min. After that, roots were transferred to a petri dish containing 5 ml of fresh testing solution. Then placed the root sample on the detection platform, and the NH_4_^+^ flux microsensor (NH_4_^+^ liquid ion exchanger: XY-SJ-NH4; NH_4_^+^ flux microsensor: XY-CGQ-01; Xuyue (Beijing) Sci. &Tech. Co., Ltd., Beijing, China.) was positioned close to the root tip (root hair zone) of cucumber seeding. The tip of NH_4_^+^ flux microsensor was about 5 μm form the root surface without touched the root. 10 min for each sample and 6 replicates per group. Use imFluxes software (imfluxes.com, Xuyue (Beijing) Sci. & Tech. Co., Ltd., Beijing, China) to obtained NH_4_^+^ flux data and process them. NO_3_^−^ flux detection steps are exactly the same as NH_4_^+^ [78].

### Estimation of total nitrogen contents

The total nitrogen (N) contents in root and shoots were estimated using the method described by Anwar et al. [75, 79].

### Endogenous EBR contents determination

EBR contents were determined using an enzyme-linked immunosorbent assay technology (ELISA) at the College of Agronomy and Biotechnology, China Agricultural University, Beijing, China [48].

### Quantitative real-time polymerase chain reaction (qRT-PCR)

Total RNA was isolated using RNAprep Pure Kit (TANGEN) and Fast Quant RT Kit (TANGEN) was used to synthesized first strand cDNA, as described by Anwar et al. [75]. Additionally, primers were designed by using Primer Premier 5 software (Additional file 1: Table S1).

### Statistical analysis

Statistix 8.1 software (www.statistix.com) was used to analyze the difference between treatments. The figures were drown by using Graphpad Prism 5 (www.graphpad.com), as described by Anwar et al. [75].

## Additional files

Additional file 1: **Table S1.** The list of specific primers for Quantitative Real-Time Polymerase Chain Reaction (qRT-PCR). (XLSX 10 kb)

## Data Availability

The supporting data are available within the article and additional files.

## Abbreviations

AMT1: Ammonium Transporter 1
ANOVA: Analysis of Variance
BES1: BRI1-EMS SUPPERSSOR 1
BZR: Brassinazole
BZR1: BRASSINAZOLE RESISTANT 1
COR: Cold Responsive Genes
EBR: 24-Epibrassinolide
GOGAT: Glutamate Synthase
GS: Glutamine Synthetase
HSP: Heat Shock Proteins
LSD: Least Significant Difference
N: Nitrogen
NH_4_^+^: Ammonium
NiR: Nitrite Reductase
NMT: Non-invasive Micro-test Technology
NO_3_^−^: Nitrate
NR: Nitrate Reductase
NRT1: Nitrate Transporter 1
RZT: Root Zone Temperature
