# Correction to: 24-Epibrassinolide promotes NO_3_^−^ and NH_4_^+^ ion flux rate and NRT1 gene expression in cucumber under suboptimal root zone temperature

**DOI:** 10.1186/s12870-019-2089-z

**Published:** 2019-10-28

**Authors:** Ali Anwar, Yansu Li, Chaoxing He, Xianchang Yu

**Affiliations:** The Institute of Vegetables and Flowers, Chinese Academy of Agricultural Sciences, Beijing, China

**Correction to: BMC Plant Biol (2019) 19: 225**

**https://doi.org/10.1186/s12870-019-1838-3**

In the original publication of this article [1], the author pointed out there is an error in Figs. 4 and 5. The ‘BR’ should be replaced by ‘EBR’. The correct figures are below:

**Fig. 4:**
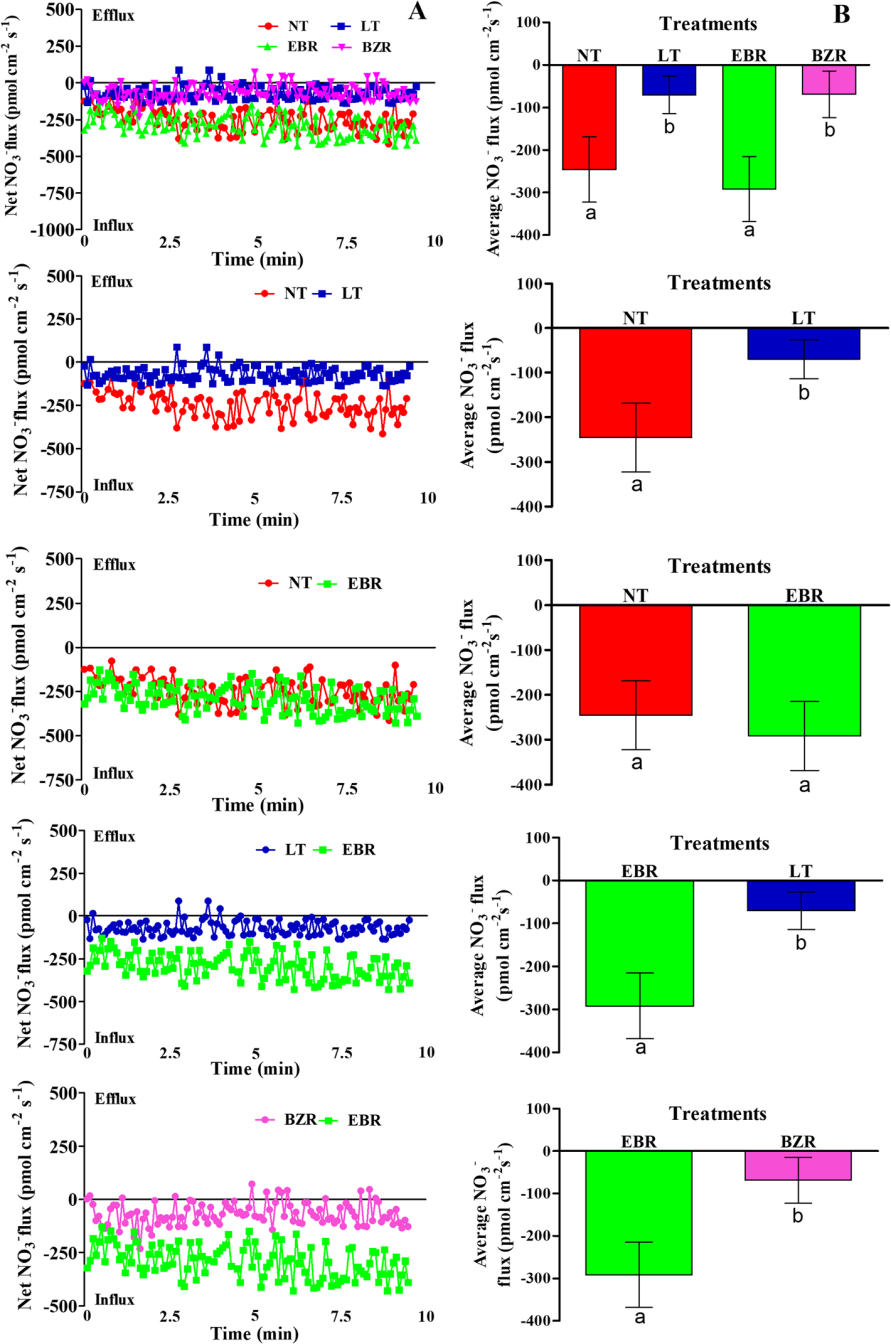
Effects of suboptimal RZT and EBR on NO_3_^−^ (A; scatter NO_3_^−^ flux rate, B; average NO_3_^−^ flux rate) flux rate in roots of cucumber seedlings under suboptimal RZT treatment. Flux rate was recorded for 10 min in roots seven days after treatment. Each point is the mean of nine individual seedlings and bars indicate standard deviations. Treatments with the same letters are not significantly different by the least significant difference (LSD) test at *P* = 0.05

**Fig. 5:**
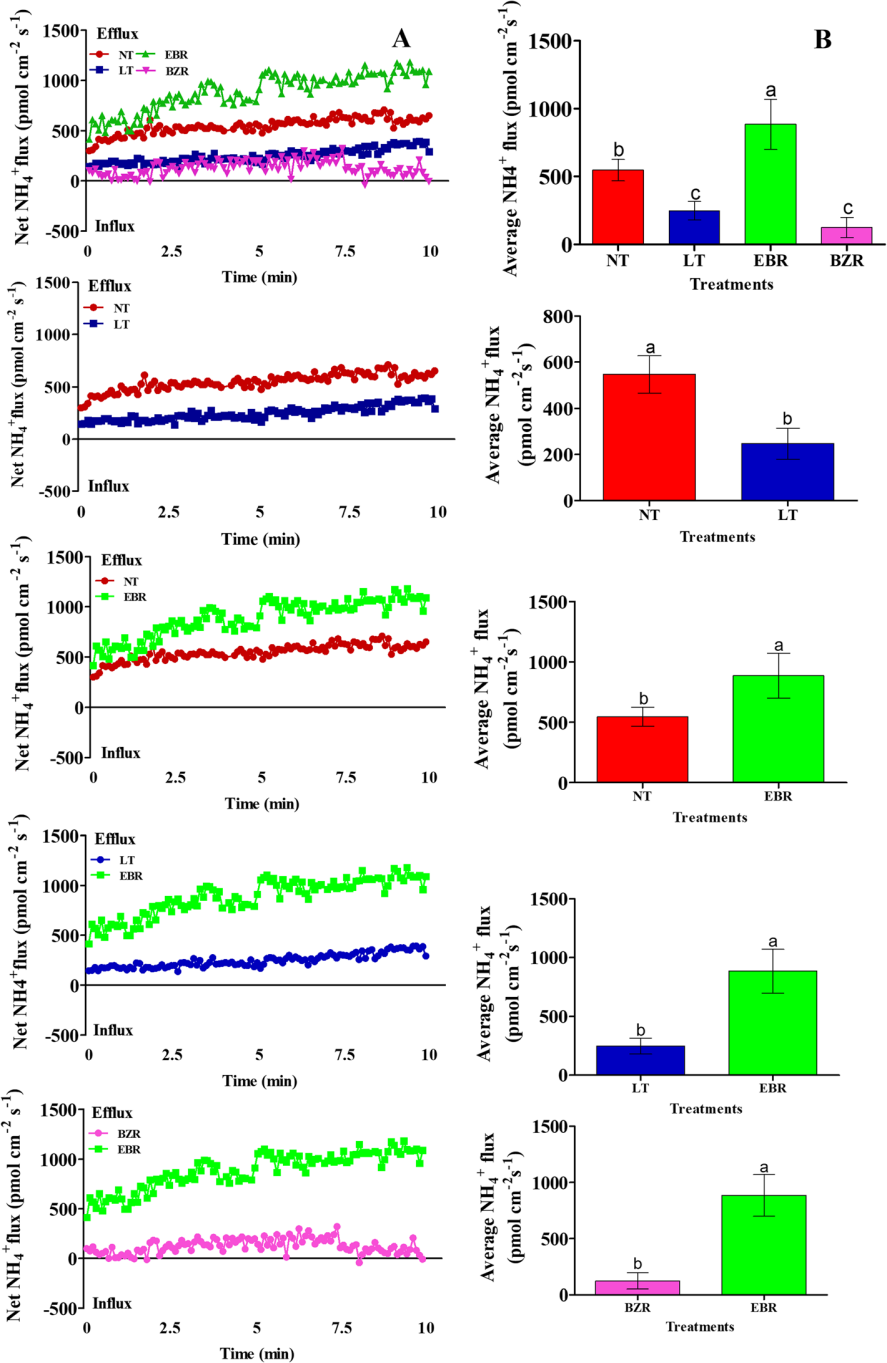
Effects of suboptimal RZT and EBR on NH_4_^+^ (A; scatter NH_4_^+^ flux rate, B; average NH_4_^+^ flux rate) flux rate in roots of cucumber seedlings under suboptimal RZT treatment. Flux rate was recorded for 10 min in roots seven days after treatment. Each point is the mean of nine individual seedlings and bars indicate standard deviations. Treatments with the same letters are not significantly different by the least significant difference (LSD) test at *P* = 0.05
